# *Selaginella magna* (Selaginellaceae), a new species from China

**DOI:** 10.3897/phytokeys.273.170753

**Published:** 2026-04-16

**Authors:** Hou-Hua Fu, Hong-Jin Wei, Yong-Jun Guo, Shuang-Quan Zheng, Shi-Pin Chen, Bin Chen

**Affiliations:** 1 Forestry College, Fujian Agriculture and Forestry University, Fuzhou, 350002, China Forestry College, Fujian Agriculture and Forestry University Fuzhou China; 2 Eastern China Conservation Centre for Wild Endangered Plant Resources, Shanghai Chenshan Botanical Garden, Shanghai, 201602, China Eastern China Conservation Centre for Wild Endangered Plant Resources, Shanghai Chenshan Botanical Garden Shanghai China; 3 State-owned Forest Farm on the Outskirts of Sanming, Sanming, Fujian, 365000, China State-owned Forest Farm on the Outskirts of Sanming Sanming China

**Keywords:** Deciduous plant, erect habit, IUCN Red List, Selaginella subg. Stachygynandrum, suborbicular sporophyll

## Abstract

*Selaginella magna* (Selaginellaceae), a new species in S. subg. Stachygynandrum from Guangxi, Fujian and Hunan, China, is here described and illustrated. It is the largest erect *Selaginella* species in China, characterized by acropetally shortening branches acropetally. *Selaginella magna* is most similar to *S. willdenowii*, but can be differentiated by its erect main stem, rhizophores restricted to rhizomes and stolons, and the absence of auricles at the base of both ventral and axillary leaves. Its triangular frond-like leafy stem with an acuminate apex confers a high degree of distinguishability in its natural habitat.

## Introduction

*Selaginella* is the only genus in the family Selaginellaceae, which comprises 700–800 species (Jermy 1990; Zhang 2004; Zhang et al. 2013; Zhou and Zhang 2015; PPG I 2016), and is widespread across all continents except Antarctica, with the highest species diversity found in tropical and subtropical regions. The genus was recently split into seven subfamilies and 19 genera by Zhou and Zhang (2023), but the classification is controversial (Valdespino et al. 2024). Recently, Zhou et al. (2025) reiterated their treatment and added a new subfamily. Here we adopt a broadly defined *Selaginella*, with infrageneric classification according to Weststrand and Korall (2016) and Zhang et al. (2024). In China, approximately 103 *Selaginella* species have been recorded (Liu et al. 2025), including *S. kraussiana* (Kunze) A. Braun, a cultivated plant native to Africa. Most Chinese species are classified within S. subg. Stachygynandrum (P. Beauv. ex Mirb.) Baker, with a few exceptions, such as *S. sibirica* (Milde) Hieron. featuring monomorphic and linear vegetative leaves, *S. remotifolia* Spring, noted for its articulate stems, and *S. sanguinolenta* (L.) Spring, a member of the *S. sanguinolenta* group which exhibits monomorphic traits and is phylogenetically resolved as the sister clade to all other members of the subgenus (Zhang et al. 2020, Tang et al. 2023, Zhang et al. 2024).

In 2020, a distinct taxon of Selaginella subg. Stachygynandrum was collected in the Xianrengu National Forest Park, central Fujian Province of China. The plant has a broad stature and is superficially similar to *S. trichoclada* Alston and *S. willdenowii* (Desv. ex Poir.) Baker in its stem surface characteristics, sporophyll shape, size and branching pattern of lager lateral branches. However, it is characterized by the branches that gradually shorten toward the apex of the main stem. If the shoot is regarded as a fern frond, its branching portions resemble a blade with a cuspidate apex, a feature distinguishing it from most other large species in Selaginellaceae. Based on morphological studies, this plant is described herein as a new species and is named *Selaginella magna*.

## Materials and methods

### Morphological studies

The putative new species, *Selaginella magna*, was collected from Fujian Province and the collections deposited in CSH, IBK, FJFC, KUN, PE, PYU, IBSC and SDFS. Both its shoots and spores (including 16 megaspores and ca. 30 microspores) were observed and photographed under Olympus SZX16 stereo microscopes. Image stacking and dimension measurements were conducted utilizing image processing Zerene Stacker software. Species in the morphological comparison included *S. helferi* Warb., *S. pseudopaleifera* Hand.-Mazz., *S. trichoclada*, and *S. willdenowii*, which share large leafy stems, similar secondary and tertiary branching pattern, entire-margined and similarly shaped trophophylls. Herbarium specimens or digital images were examined from the CSH, CVH (https://www.cvh.ac.cn/), GBIF (https://www.gbif.org/) and JSTOR (https://plants.jstor.org/). Morphological characters for comparative species were mainly based on Zhang (2004) and Zhang et al. (2013). Furthermore, to identify additional distribution records of *S. magna* in China, we investigated two invaluable online resources containing large collections of photos of Chinese plants: The Chinese Field Herbarium (**CFH**, https://www.cfh.ac.cn/) and the Plant Photo Bank of China (**PPBC**, https://ppbc.iplant.cn/).

## Results

Examination of material from CSH, CVH, CFH and PPBC revealed additional morphologically similar specimens from Hunan, Jiangxi, Guangdong, and Guangxi (China), which had previously been misidentified as other species, for instance, *S. helferi*, *S. decipiens* Warb. and *S. delicatula* (Desv. ex Poir.) Alston. These findings imply a broader geographic range than was previously comprehended, as it was formerly considered to be confined solely to a particular spot within Fujian province.

The morphological comparisons between *Selaginella magna* and its similar species are shown in Table 1. *Selaginella magna* is easily distinguished from *S. pseudopaleifera* and *S. trichoclada* by its glabrous stems. The latter two have pubescent stems and triangular auricles on the acroscopic base of the ventral leaves. Among the four species, *S. magna* most closely resembles *S. willdenowii* in having suborbicular sporophylls and glabrous stems. However, *S. magna* differs in growth habit, dimension of ventral leaves, basal form of ventral and axillary leaves, branching pattern and leaf margin texture.

**Table 1.** Morphological comparison of *Selaginella magna* with closely similar species in China.

Characters		*S. magna*	*S. willdenowii*	*S. helferi*	*S. pseudopaleifera*	*S. trichoclada*
Life habit		seasonally green	evergreen	evergreen	evergreen	evergreen or seasonally green
Tall/length (cm)		60–75 or more	100–200 or more	50–200 or more	50–100	45–80(–110)
Growth habit		erect	scandent	scandent	ascending	erect
Rhizophores	surface	glabrous	with some spinelike protuberances at base	with some spinelike protuberances at base	glabrous	glabrous
	growth site	confined to base of main stem and creeping rhizomes and stolons	often extending to upper part of main stem	extending to middle part of main stem	confined to creeping rhizomes and stolons	confined to base of stem
Main stem	sinuosity	not or slightly zigzag	not or slightly zigzag	somewhat zigzag	not or somewhat zigzag	obviously zigzag
	surface	glabrous	mostly glabrous	glabrous	glabrous	pubescent
Primary leafy branches	relative length	gradually shorten towards apex of main stem	similar length below apex of main stem	similar length below apex of main stem	similar length below apex of main stem	similar length below apex of main stem
	stem surface	glabrous	nearly glabrous	glabrous	pubescent	pubescent
Ventral leaves	acroscopic base	without auricle	with rounded auricle	with rounded auricle	with triangular auricle	with triangular auricle
	dimension (mm)	2–4.8 × 0.8–2.6	2.8–4 × 1–1.5	2.3–4.2 × 0.9–1.8	3–3.6 × 1–1.5	2.5–4 × 0.8–1.4
Axillary leaves	base form	obtuse	biauriculate	biauriculate	biauriculate	biauriculate
Sporophylls	shape	suborbicular	suborbicular	ovate-lanceolate	ovate-lanceolate	suborbicular
	membranous edges	without	with	with	without	with

### Taxonomical treatment

#### Selaginella magna

Taxon classificationPlantaeSelaginellalesSelaginellaceae

H.J.Wei sp. nov.

B96738A5-B8F6-5783-A966-D382BA61BD07

urn:lsid:ipni.org:names:77378700-1

Figs 1, 2

##### Type.

China • Fujian: Sanming City, Sanyuan District, Sanming Xianrengu National Forest Park, Hutou Mountain, Dake Waterfall Group, in a valley, 26°14'55"N, 117°40'27"E, elev. 700–750 m, 9 November 2025, *She-Lang Jin JSL10756* (holotype: CSH0200008; isotypes: CSH!, IBK!, FJFC!, KUN!, PE!, PYU!, IBSC!, SDFS!).

**Figure 1.**
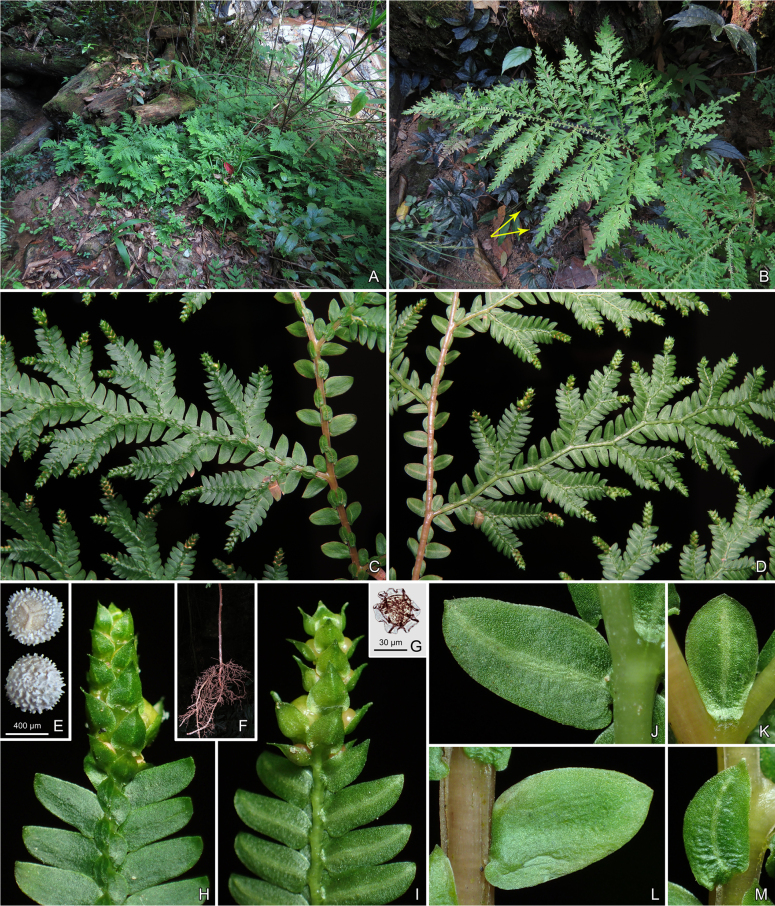
*Selaginella magna*. **A**. Habitat; **B**. Habit (arrows indicating portion of plant shown in line drawing); **C**. Abaxial view of portion of plant; **D**. Adaxial view of portion of plant; **E**. Megaspores; **F**. Lower portion of main stem with stolons; **G**. Microspore; **H**. Abaxial view of strobile; **I**. Adaxial view of strobile; **J**. Adaxial view of ventral leaf with portion of stem; **K**. Axillary leaf; **L**. Abaxial view of ventral leaf with portion of stem; **M**. Dorsal trophophyll.

**Figure 2.**
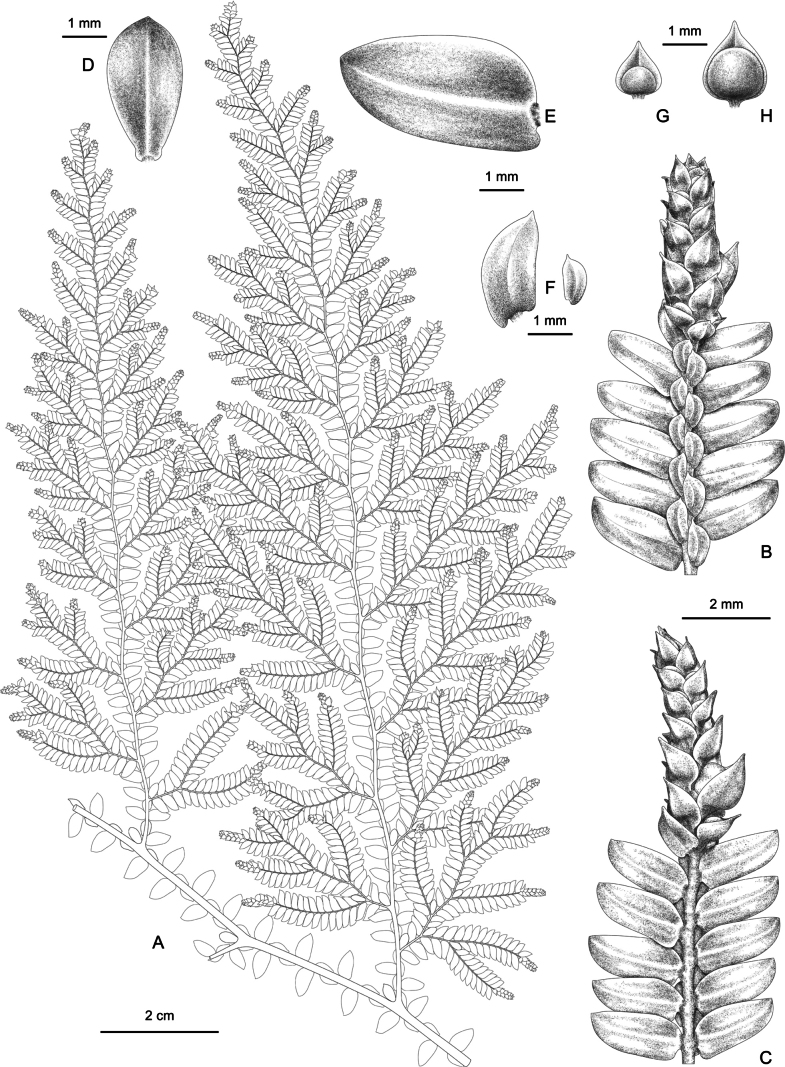
*Selaginella magna* (based on paratype *JSL7741* in CSH). **A**. Portion of plant (see Fig. 1B); **B**. Abaxial view of strobile; **C**. Adaxial view of strobile; **D**. Axillary leaf on main stem; **E**. Ventral leaf on main stem; **F**. Dorsal leaves on main stem (left) and ultimate branchlet (right); **G**. Microsporophyll; **H**. Megasporophyll. Illustrated by Hong-Jin Wei.

##### Diagnosis.

*Selaginella magna* is closely related to *S. willdenowii*, but differs by its rhizophores being glabrous (compared to spine-like protuberances at base in the latter), confined to creeping rhizomes and the base of the stem (vs. often extending to the upper part of the main stem in the latter), branches that progressively shorten toward the apex (vs. nearly equal in length except young branches at the apex), ventral and axillary leaves without auricles (vs. with rounded auricles) at the base, and sporophylls that are not white-margined (vs. obviously white-margined).

##### Description.

**Plants** terrestrial, seasonally green, erect, 60–75 cm or more, with creeping subterranean rhizome and stolons; rhizome protostelic. **Rhizophores** restricted to base of stem and creeping rhizomes and stolons, 2–7 cm long, slender, 0.15–0.20 mm in diam., roots much forked, nearly glabrous. **Main stem** branched from lower part, pinnately branched, straight, or sometimes slightly zigzag, reddish when fresh, nonarticulate, stramineous when dry, unbranched main stem 5.5–19.0 cm tall; 2.0–3.3 mm in diam. in lower part, terete, sulcate, glabrous, with 3 vascular bundles arranged in a single row, fertile stems 2–3 times pinnately branched, main stem together with leaves 3.0–5.7 mm wide at middle; primary leafy branches 7–9 pairs, larger ones 24–30 × 8–26 cm, usually 3 times pinnately branched, basal two adjacent primary branches on main stem 5–9(–15) cm apart, tertiary branches simple or forked, occasionally once pinnately branched, branchlets sparse and regular, ultimate branches along with leaves 3–7 mm wide. **Leaves** herbaceous. **Axillary leaves** symmetrical, base obtuse or slightly biauriculate, margin entire, obovate on main stem, obovate to oblong-elliptic on branches, those on main stem obviously larger than those on branches, 1.4–3.4 × 0.7–2.5 mm. **Dorsal leaves** asymmetrical, those on branches approximate to imbricate, overlapping at leaf apex, falcate-oblong, sometimes nearly falcate-obovate; dorsal leaves on main stem larger than those on branches, 0.8–3.5(–4.0) × 0.4–1.8 mm, not carinate, base obliquely subcordate, slightly uniauriculate at basiscopic side, margin entire, apex obtusely cuspidate. **Ventral leaves** asymmetrical, spreading or slightly ascending, those on main stem distant below apex, oblong-ovate, and those on branches distant to overlapping, oblong-ovate to falcate-oblong; ventral leaves on main stem obviously larger than those on branches, 2.0–4.8 × 0.8–2.6 mm, margin entire, apex obtuse; acroscopic base rounded, not overlapping stem and branches; basiscopic base rounded or rounded truncate, sometimes slightly enlarged and subauriculate, not or slightly overlapping stem and branches. **Strobili** solitary, terminal, compact, tetragonal, 2–6(–8) × 1.2–1.6(–2.6) mm; sporophylls uniform, not white-margined, occasionally with 1 cell wide, not obviously membranous margin, broadly ovate or suborbicular, carinate, margin entire, base shallowly cordate, apex acute or cuspidate; microsporophyll up to 1.4 × 1.2 mm or more, megasporophylls often larger, up to 1.8 × 1.6 mm, distributed in middle on lower side of strobilus; microsporangia semicircular, brown, 0.7–0.8 × 0.5–0.6 mm, cells regular, megasporangia suborbicular, brownish green or pale green, 1.0–1.2 mm in diam..

##### Spore morphology.

Megaspores white or yellowish white, four per sporangium, orbicular or suborbicular in polar view, oblate spheroid in equatorial view, 320–400 μm in polar axis and (290–)400–600 μm in equatorial axis; prominent laesurae extend 5/9–2/3 of the distance to the equator in polar view, both proximal and laesurae surfaces covered with granulate and verrucate elements, other surfaces with abundant, shot baculate and discrete or coalescent tubercles (Fig. 1E). Microspores are yellowish orange, suborbicular in polar view, 22–27 μm in polar axis, perispores clothed with long, curved lamellate ornamentations (Fig. 1G).

##### Distribution and habitat.

**Figure 3.**
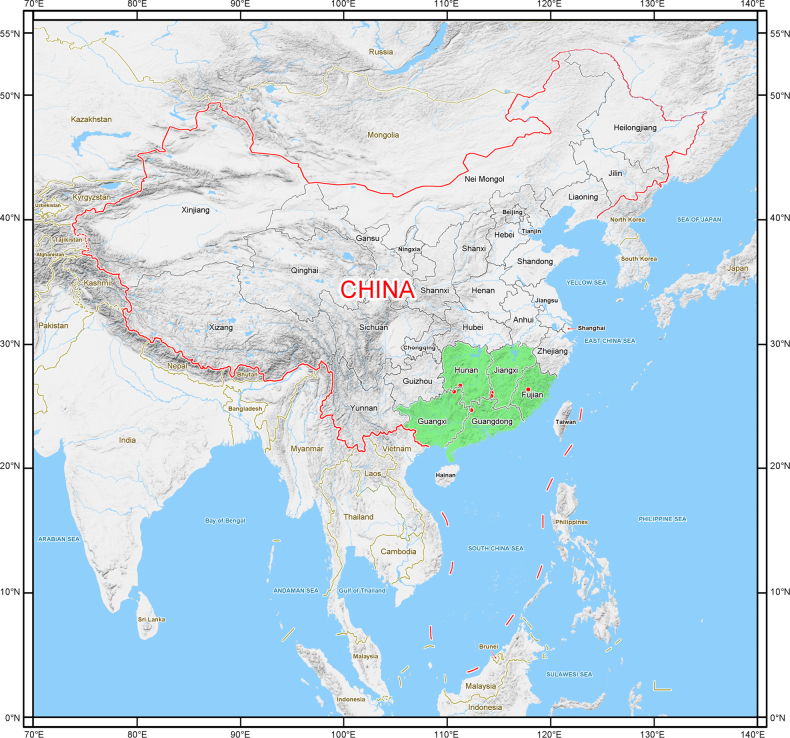
Geographical distribution of *Selaginella magna* (red dots) in China.

*Selaginella magna* was found in at least seven localities within China’s mid-subtropical zone at similar latitudes, with elevations ranging from 600 to 1076 meters. These localities include northeastern Guangxi, northwest Guangdong, central Fujian, southwest Hunan, and southwest Jiangxi (Fig. 3). Populations in Fujian and Guangxi inhabit valley lowlands, while one population in Hunan thrived in a bamboo-broadleaved mixed forest.

##### Conservation assessments and ecology.

At least nine populations were found in China, two of which were separated by only a few hundred meters. The combined range of these populations extends roughly 720 kilometers east-west and 250 kilometers north-south, encompassing a total area of approximately 82,600 square kilometers, as defined by the outermost occurrence points. The plant typically forms dense, compact colonies through localized vegetative growth of its horizontally spreading rhizomes. *Selaginella magna* may occur in other areas. As its broader distribution has not yet been fully investigated, we recommend that it be classified as Data Deficient (DD) under the IUCN Red List criteria (IUCN 2024).

##### Etymology.

The epithet of the new species is derived from the Latin “*magna*”, meaning great or large.

##### Chinese name.

硕大卷柏 (shuò dà juăn băi).

##### Paratypes.

The same place as the holotype, 31 December 2020, *She-Lang Jin & Bin Chen JSL7741* (CSH!)·ibid., 8 July 2021, *She-Lang Jin & Bin Chen JSL8163* (CSH!). Shanghai Municipality, Songjiang District, Shanghai Chenshan Botanical Garden, cultivated (introduced by from the type locality), 10 November 2025, She-Lang Jin *JSL10440A* (CSH!).

##### Additional specimens examined.

China • Hunan Province, Dong’an County, Shunhuangshan, 19 September 2012, *She-Lang Jin & Hui Shang JSL-174* (CSH0039784, CSH0039785, CSH0039786, CSH003977)·ibid., elev. 750 m, 4 October 2017, *Zhong-Long Zhu & Hui Li 0131* (HUST 00019848) • Hunan Province, Xinning County, Shunhuangshan, elev. 900 m, 3 October 2017, *Zhong-Long Zhu & Hui Li 0302* (HUST 00019846) • Jiangxi Province, Chongyi County, Qiyunshan Nature Reserve, elev. 600 m, 23 July 2007, *Yue-Hong Yan, Xi-Le Zhou & Lan-Ying Wang 4042* (HUST 00003748, PE 01780752) • Jiangxi Province, Chongyi County, Fengzhou Town, Changlong’ao, elev. 1,000 m, 8 July 2007, *Lan-Ying Wang W.163* (HUST 00019847) • Guangxi Zhuang Autonomous Region, Guilin City, Maoershan National Nature Reserve, Taozitou Valley, elev. 638 m, 24 October 2016, *Xi-Le Zhou, Jing Liu, Chun-Yu Zou & Jiu-Bing Zhang ZXL07637* (CSH0138900) • Guangdong Province, Liannan County, Xiangping Town, Paidu village, Ma’an Group, elev. 1,076 m, 30 May 2024, *Hong-Lin Zhang, Tong Zhu et al. DN165* (SWI!).

## Discussion

*Selaginella magna* exhibits a combination of tetrastichous phyllotaxy in both vegetative leaves and sporophylls, nonarticulate stems, rhizophores restricted to creeping protostelic rhizomes and the base of the main stem, and megaspores bearing thickened laesurae and solitary protrusions. These characteristics suggest that it belongs to S. subg. Stachygynandrum (sensu Weststrand and Korall 2016).

*Selaginella magna* and *S. willdenowii* are morphologically similar, particularly in stem surface characteristics, leaf shape and leaf size. However, *S. magna* is an erect plant, typically less than 1 meter tall, with rhizophores confined to the stem base and creeping subterranean rhizomes, whereas *S. willdenowii* is a scandent plant, reaching 2 meters or more in length, with rhizophores arising along the main stem. Therefore, these two species can be distinguished from each other in the field by their growth habit. In addition, *S. magna* is a deciduous plant, with its shoots dying back in winter, and it inhabits middle subtropical zones. While *S. willdenowii* is an evergreen plant and occurs in southern subtropical to tropical zones.

*Selaginella trichoclada*, an erect plant primarily distributed in mid-subtropical areas and overlapping with the range of the novel species, shares similar leaf shapes and dimensions in both vegetative leaves and sporophylls. It is easily identified by its notably pubescent stems and ventral leaves with a distinct triangular auricle at the acroscopic base.

Within Selaginellaceae, some erect species often exhibit a branching pattern where the primary branches gradually shorten towards the tip of the main stem, or are sometimes abruptly reduced in the upper portion, resulting in an acuminate or cuspate apex to the overall plant outline, for example, *S. hoffmannii* Hieron., *S. umbrosa* Lem. ex Hieron. and *S. moellendorffii* Hieron. This feature is sometimes helpful for rapid species identification in the field. *Selaginella fulcrata* (Buch.-Ham. ex D. Don) Spring is another large-sized erect plant occurring in Nepal and India (Shalimov et al. 2019). Its gross morphology is somewhat similar to that of the new species, including growth habit, branching pattern, leaf packing density and sporophyll shape. However, it features ciliate ventral vegetative leaves.

*Selaginella magna* possesses distinctive diagnostic features that allow for clear differentiation, even when specimens are incomplete. Key characteristics include: glabrous stems, trophophyll with entire margins lacking distinct membranous edges, and the absence of auriculate appendages at the base of ventral and axillary leaves. Importantly, *S. magna* has larger ventral leaves (see Table 1) and displays a more densely arranged ultimate ramification pattern compared to the sparser arrangement observed in morphologically similar species. The differences in leaf size and ramification density provide additional distinguishing characteristics, useful when consulting digitized specimens or when trying to identify from field photographs with bad resolution. Within Selaginellaceae in China, this species represents the largest erect species of *Selaginella* known to date with a triangular or ovate-triangular “frond” (when considering the entire branching system as a frond). These pronounced morphological distinctions allow for unambiguous differentiation from congeners, facilitating field identification. Furthermore, preliminary phylogenetic analyses based on combined plastid genome regions (*rbcL* & ITS) also supported *S. magna* as a distinct species, thereby reinforcing its taxonomic validity and underscoring the value of molecular evidence for *Selaginella* differentiation. Future studies could include additional molecular markers to further elucidate the phylogenetic relationships within this group.

## Supplementary Material

XML Treatment for Selaginella magna

## Additional information

### Use of AI

No use of AI was reported.

### Author ORCIDs

Hou-Hua Fu https://orcid.org/0009-0001-6905-4280

Hong-Jin Wei https://orcid.org/0000-0002-3679-6683

Yong-Jun Guo https://orcid.org/0009-0005-9181-381X

Shuang-Quan Zheng https://orcid.org/0009-0008-8875-0290

Shi-Pin Chen https://orcid.org/0000-0002-8090-6616

Bin Chen https://orcid.org/0000-0003-1822-4486

### Data availability

All of the data that support the findings of this study are available in the main text.
